# Establishment of epidemiological cut-off values of cefotaxime-sulbactam against *Escherichia coli*, *Klebsiella pneumoniae*, *Enterobacter cloacae*, *Proteus mirabilis*, *Serratia marcescens*, *Acinetobacter baumannii* and *Staphylococcus aureus*

**DOI:** 10.3389/fmicb.2026.1866137

**Published:** 2026-09-02

**Authors:** Menglan Zhou, Yun Wu, Wei Kang, Qiwen Yang, Bo Zheng, Fupin Hu, Yunsong Yu, Chao Zhuo, Ge Zhang, Jingjia Zhang, Jin Li, Tong Wang, Haotian Gao, Yingchun Xu

**Affiliations:** 1 Department of Laboratory Medicine, Peking Union Medical College Hospital, Chinese Academy of Medical Sciences and Peking Union Medical College, Beijing, China; 2 Institute of Clinical Pharmacology, Peking University, Beijing, China; 3 Huashan Hospital of Fudan University, Shanghai, China; 4 Zhejiang Provincial People’s Hospital, People’s Hospital of Hangzhou Medical College, Hangzhou, China; 5 Department of Infectious Disease, The First Affiliated Hospital of Guangzhou Medical University, Guangzhou, China

**Keywords:** *acinetobacter baumannii*, cefotaxime, cefotaxime-sulbactam, ECOFF, epidemiological cut-off

## Abstract

**Objective:**

To establish the epidemiological cut-off (ECOFF) value of cefotaxime-sulbactam against *Escherichia coli*, *Klebsiella pneumoniae*, *Enterobacter cloacae*, *Proteus mirabilis*, *Serratia marcescens*, *Acinetobacter baumannii*, and *Staphylococcus aureus*.

**Methods:**

A total of 1,770 non-duplicate clinical isolates were collected from five laboratories in four cities in China. Minimum inhibitory concentrations (MICs) of cefotaxime-sulbactam were determined independently using the broth microdilution method according to EUCAST standards. For *A. baumannii*, cefotaxime-sulbactam was tested at a 2:1 ratio, whereas for the other species sulbactam was tested at a fixed concentration of 4 mg/L. ECOFFs were determined using ECOFFinder software and visual estimation following EUCAST principles.

**Results:**

Statistical endpoints at 95%, 97.5%, 99%, 99.5%, and 99.9% were calculated. The final ECOFFs were assigned based on concordance between ECOFFinder-derived estimates and visual inspection, using the 99% endpoint as the primary statistical estimate. The ECOFFs were 0.25/4 mg/L for *E. coli* and *K. pneumoniae*; 0.5/4 mg/L for *E. cloacae*; 0.06/4 mg/L for *P. mirabilis*; 1/4 mg/L for *S. marcescens*; 4/4 mg/L for *S. aureus*; and 4/2 mg/L for *A. baumannii*.

**Conclusion:**

ECOFFs of cefotaxime–sulbactam were established for seven clinically important bacterial species. These results provide essential reference values for distinguishing wild-type from non-wild-type isolates and support future breakpoint-setting and antimicrobial resistance surveillance.

## Introduction

Cefotaxime is a third-generation cephalosporin antibiotic with broad-spectrum activity against Gram-negative and Gram-positive bacteria, including aerobes and certain anaerobic pathogens. It is widely used to treat infections involving the lower respiratory tract, genitourinary system, central nervous system, and for prophylactic measure against postoperative infections (Todd and Brogden, 1990). Sulbactam is an irreversible, competitive β-lactamase inhibitor that exhibits potent activity against β-lactamases produced by *Staphylococcus aureus* and most Gram-negative bacilli. In addition to its β-lactamase inhibitory properties, sulbactam displays intrinsic antibacterial activity against *Bacteroides* spp. and *Acinetobacter* spp (Akova, 2008). Cefotaxime-sulbactam, a combination of cefotaxime and sulbactam, is commonly used to treat moderate to severe infections caused by β-lactamase-producing bacteria. It was widely used in the Indian region (Gondane and Pawar, 2023), China (Zheng and Xu, 2024), and Germany (Kaur et al., 2014; Manncke et al., 1996).

Importantly, it has also been considered safe for pediatric use, with clinical studies demonstrating favorable therapeutic efficacy and acceptable safety profiles in pediatric populations (Pareek et al., 2008; Readman et al., 2023). Moreover, compared with newer antimicrobial agents or combination regimens, cefotaxime-sulbactam offers a cost advantage (Kaur et al., 2014), thereby reducing treatment-related financial burden.

Despite its widespread clinical use, epidemiological cut-off values (ECOFFs) or clinical breakpoints for cefotaxime-sulbactam have not been established yet. In routine practice, susceptibility results are often interpreted by reference to cefotaxime breakpoints alone, although the validity of this approach for the combination remains uncertain. Consequently, interpretation of antimicrobial susceptibility testing (AST) results for cefotaxime-sulbactam is challenging and potentially unreliable. Establishing ECOFFs for cefotaxime–sulbactam would therefore help distinguish wild-type (WT) from non-wild-type (NWT) isolates in the absence of interpretive criteria.

ECOFFs represent the highest MIC values that differentiate WT isolates, which lack acquired resistance mechanisms, from NWT isolates that harbor resistance mechanisms and may exhibit reduced susceptibility. According to EUCAST, ECOFFs are typically defined as the 99th percentile of the phenotypically WT population (EUCAST, 2021). The standard approach requires uncensored MICs and assumes that the phenotypically WT population can be readily identified, either because the population has been minimally exposed to the drug or because the MIC distribution is strongly bimodal with no clear separation between WT and non-WT. These conditions are challenging to satisfy, particularly when the study cohort includes a substantial proportion of drug-resistant isolates, resulting in complex MIC distributions that preclude the straightforward identification of a pure phenotypically WT population. To reduce distortion of WT distributions, some studies apply predefined microbiological exclusion criteria to remove isolates likely to carry major resistance mechanisms (CRyPTIC Consortium, 2022). In the present study, given that cefotaxime-sulbactam lacks activity against strains producing AmpC β-lactamases and carbapenemases, isolates suspected of harboring these enzymes were excluded based on their susceptibility profiles to cefoxitin and carbapenems.

The objective of this study was to determine the ECOFFs of cefotaxime-sulbactam against seven species: *E. coli, K. pneumoniae, E. cloacae, S. marcescens, P. mirabilis, A. baumannii*, and *S. aureus*. We included 1170 isolates collected from five different laboratories across four cities in China, and ultimately the MIC were determined based on standardized EUCAST methodologies.

## Materials and methods

### Isolate collection and quality control strains

A total of 1,770 non-duplicate clinical isolates were collected from five tertiary hospitals between 2017 and 2020 in China. Participating institutions were Huashan Hospital, Fudan University (Shanghai, Laboratory 1), Peking Union Medical College Hospital (Beijing, Laboratory 2), Peking University First Hospital (Beijing, Laboratory 3), Sir Run Run Shaw Hospital, Zhejiang University (Hangzhou, Laboratory 4), and Tongji Hospital, Huazhong University of Science and Technology (Wuhan, Laboratory 5).

To enrich for isolates likely representing WT or near-WT populations, predefined species-specific phenotypic selection criteria were applied. For *E. coli, K. pneumoniae, and P. mirabilis*, isolates were required to be susceptible to cefoxitin and negative for carbapenemase production. For *E. cloacae* and *S. marcescens*, cefoxitin susceptibility was not used as a selection criterion because of their chromosomal AmpC background and expected cefoxitin-resistant phenotype; isolates were required to be susceptible to ceftazidime and carbapenems. For *A. baumannii*, isolates were required to be susceptible to carbapenems. For *S. aureus*, only methicillin-susceptible *S. aureus* (MSSA) isolates were included.

To enrich for isolates likely representing WT or near-WT populations, predefined phenotypic selection criteria were applied. For *E. coli, K. pneumoniae*, and *P. mirabilis*, isolates were required to be susceptible to cefoxitin and negative for carbapenemase production. For *E. cloacae* and, isolates were required to be susceptible to ceftazidime and negative for carbapenemase production. The rationale for the different criterion used for *E. cloacae* was its intrinsic inducible AmpC background, which limits the interpretability of cefoxitin susceptibility.

Isolates were consecutively and randomly chosen from a strain repository meeting the above criteria without imposing additional selection conditions to avoid selection bias. All non-duplicate isolates were obtained from a wide range of clinical specimens, including blood, sputum, urine, secretions, pus, wounds, ulcers, swabs, drainage fluids, tissues, and other body fluids. Species identification was confirmed using MALDI-TOF MS. The numbers of isolates included for each species were as follows: *E. coli* (*n* = 252), *K. pneumoniae* (*n* = 256), *E. cloacae* (*n* = 252), *P. mirabilis* (*n* = 252), *S. marcescens* (*n* = 252), *S. aureus* (*n* = 255), and *A. baumannii* (*n* = 251), yielding a total of 1,770 isolates. Three QC strains were *E. coli* ATCC25922, *A. baumannii* NCTC13304 and *S. aureus* ATCC29213.

### Antimicrobial susceptibility test

#### Blind sample external quality control (EQC) in five labs

Prior to clinical isolate testing, 14 external quality control (EQC) samples (two high-MIC and two low-MIC isolates per species) and three ATCC reference strains (*E. coli* ATCC 25922, *A. baumannii* NCTC13304, and *S. aureus* ATCC 29213) were tested in each laboratory. MIC testing for QC strains was performed daily for three consecutive days. MIC results were considered valid only when QC values fell within acceptable ranges. For laboratories other than the central laboratory (Peking Union Medical College Hospital, Laboratory 2), EQC results were accepted only when at least two out of three results fell within one twofold dilution of those obtained by the central laboratory. If discrepancies were observed, re-evaluation was performed until acceptable inter-laboratory consistency was achieved. Moreover, the MIC of cefotaxime was also tested alone for comparison.

#### MIC determination for clinical isolates

The central laboratory prepared AST panels using standard powders of cefotaxime and sulbactam (National Institutes for Food and Drug Control, China) and cation-adjusted mueller-hinton broth (CAMHB) (Becton, Dickinson and Company, USA). MICs were determined using the broth microdilution method in accordance with EUCAST guidelines (ISO: 20776-1) (ISO, 2019). MIC values were read after 18 h of incubation and defined as the lowest concentration completely inhibiting visible growth. If significant growth was observed in all drug concentration wells, it was designated as > 256 mg/L.

Different cefotaxime-sulbactam testing configurations were used for *A. baumannii* and for the other species because the role of sulbactam differs biologically between these organisms. For *E. coli*, *K. pneumoniae*, *E. cloacae*, *P. mirabilis*, *S. marcescens*, and *S. aureus*, sulbactam was used at a fixed concentration of 4 mg/L, with cefotaxime concentrations ranging from 0.004 to 256 mg/L. The use of sulbactam at a fixed concentration of 4 mg/L is in strict accordance with the standardized methodology endorsed by EUCAST (EUCAST, 2026) for testing β-lactam/β-lactamase inhibitor combinations (e.g., ceftazidim-avibactam (Sader et al., 2024), ceftolozane-tazobactam and cefepime-taniborbactam (Belley et al., 2025). In contrast, for *A. baumannii*, cefotaxime-sulbactam was tested at a 2:1 cefotaxime: sulbactam ratio, with concentrations ranging from 0.004/0.002 to 256/128 mg/L. This species-specific approach was used because sulbactam has intrinsic antibacterial activity against *Acinetobacter* spp. (Lee et al., 2017; Penwell et al., 2015). The 2:1 ratio was chosen to simulate the clinically achievable peak serum concentration ratio of these two agents based on their pharmacokinetic profiles (Mouton et al., 2012). Accordingly, the ECOFF derived for *A. baumannii* should be interpreted as a cefotaxime-sulbactam combination ECOFF under the 2:1 ratio testing condition. It should not be directly compared with the fixed-sulbactam ECOFFs derived for the other species without considering this methodological difference.

#### Whole genome sequencing, assembly, and analysing

Among the 118 *P. mirabilis* isolates with MICs above the established ECOFF, 96 isolates with available viable stocks and qualified DNA were subjected to WGS to explore potential antimicrobial resistance mechanisms associated with elevated cefotaxime-sulbactam MICs. Genomic DNA was isolated from fecal-derived *K. oxytoca* strains with the TIANamp Bacteria DNA Kit (DP302). Following inoculation in LB medium (Sangon Biotech, China), the bacteria were grown at 37 ° C for 18 h. DNA purity and concentration were assessed using a NanoDrop 2000 and a Qubit 2.0 Fluorometer. Whole-genome sequencing was performed on the Illumina NovaSeq 6000 platform with 150-bp paired-end reads. Raw reads were quality-trimmed with Trimmomatic v0.38 (Bolger et al., 2014) and assembled *de novo* using Shovill v1.0.1^1^, using a minimum contig length of 200 bp. Antimicrobial resistance determinants (Alcock et al., 2020) were annotated by aligning the assembled genomes to the Comprehensive Antibiotic Resistance Database (version 3.2.3) (Liu et al., 2022), respectively.

### Data analysis

MIC distributions were reviewed by species and by laboratory before aggregation. Aggregated distributions were considered acceptable for ECOFF analysis when the modal MICs of putative WT distributions from individual laboratories were within one doubling dilution of the overall modal MIC, in accordance with EUCAST SOP 10.2.

ECOFFs were estimated using two complementary approaches: visual inspection of the MIC distributions and statistical modelling with ECOFFinder (downloaded from the EUCAST)^2^. WT upper limits corresponding to the 95%, 97.5%, 99%, 99.5%, and 99.9% percentiles were calculated. The 99% endpoint from ECOFFinder was used as the primary statistical estimate, and the final ECOFF was assigned only when it was concordant with visual inspection or differed by no more than one doubling dilution, consistent with EUCAST principles (EUCAST, 2021).

### Ethics approval

The study was approved by the Health Research Ethics Board of Peking Union Medical College Hospital (no. HS-2762).

## Results

### MIC distributions and mode MIC

In the aggregated results from the five laboratories, the MIC range of cefotaxime-sulbactam against *E. coli* and *P. mirabilis* is 0.016/4 to > 256/4 and 0.004/4 to > 256/4, with a mode of 0.03/4 mg/L and 0.016/4 mg/L respectively. For *K. pneumoniae*, the MIC values of cefotaxime-sulbactam ranged from 0.008/4 to > 256/4, with a mode of 0.03/4 mg/L. For *A. baumannii*, the MIC range of cefotaxime-sulbactam was 0.5/0.25 to > 256/128 mg/L, with a mode of 2/1 mg/L. Both *E. cloacae* and *S. marcescens* showed the same mode MIC 0.125/4 mg/L, with MIC range from 0.016/4 to > 256/4, and from 0.032/4 to 256/4, respectively. For *S. aureus*, the MIC values of cefotaxime-sulbactam ranged from 0.25/4 to > 256/4 mg/L, with a mode of 2/4 mg/L (Detailed in Tables 1, 2). Across the five centers, modal MICs of the putative WT distributions were within one doubling dilution for each species, supporting aggregation of the MIC data according to EUCAST SOP 10.2.

**TABLE 1:** Aggregated MIC distributions of cefotaxime-sulbactam for the seven study species across five participating laboratories.

Units	Number of isolates with MIC (mg/L) of:																		Total number	Mode WT MIC
	0.004/ 4	0.008/ 4	0.016/ 4	0.03/4	0.06/ 4	0.125/ 4	0.25/4	0.5/4	1/4	2/4	4/4	8/4	16/4	32/4	64/4	128/4	256/4	> 256/ 4		
Eco																				
Lab 1	1	0	0	7	4	4	1	0	1	1	7	3	5	3	4	4	3	2	50	0.03/4
Lab 2	0	0	0	5	13	4	5	1	0	1	5	3	0	5	3	3	2	2	52	0.06/4
Lab 3	0	1	7	22	9	1	3	1	1	0	1	1	1	0	0	2	0	0	50	0.03/4
Lab 4	0	0	1	3	5	4	1	0	1	7	7	2	8	4	4	1	1	1	50	0.06/4
Lab 5	0	0	6	12	8	5	0	0	1	1	1	2	2	2	5	2	1	2	50	0.03/4
Total	1	1	14	49	39	18	10	2	4	10	21	11	16	14	16	12	7	7	252	0.03/4
Kpn																				
Lab 1	0	0	0	9	15	4	3	1	0	0	0	0	0	4	0	8	5	1	50	0.06
Lab 2	0	0	1	5	31	10	2	1	1	0	1	0	2	0	1	1	0	0	56	0.06
Lab 3	0	3	12	24	5	1	1	1	0	1	0	0	1	0	0	0	1	0	50	0.03
Lab 4	0	1	1	15	5	6	2	0	0	2	1	1	0	3	2	4	1	6	50	0.03
Lab 5	0	0	3	20	14	3	0	1	1	0	0	0	4	0	2	1	1	0	50	0.03
Total	0	4	17	73	70	24	8	4	2	3	2	1	7	7	5	14	8	7	256	0.03
Ecl																				
Lab 1	0	0	0	0	7	29	7	5	1	0	0	0	0	0	0	1	0	0	50	0.125
Lab 2	0	0	0	0	3	15	23	7	3	1	0	0	0	0	0	0	0	0	52	0.25
Lab 3	0	0	0	2	9	15	10	2	3	2	0	1	0	1	2	1	0	2	50	0.125
Lab 4	0	0	0	0	4	17	10	5	3	2	3	2	2	0	1	0	0	1	50	0.125
Lab 5	0	0	1	0	6	14	12	8	1	2	1	3	0	0	1	1	0	0	50	0.125
Total	0	0	1	2	29	90	62	27	11	7	4	6	2	1	4	3	0	3	252	0.125
Pmi																				
Lab 1	1	12	12	0	5	0	0	0	1	1	0	4	0	0	1	4	1	8	50	0.008 OR 0.016
Lab 2	0	6	11	1	2	2	0	0	0	0	0	1	0	0	0	0	4	25	52	0.016
Lab 3	0	2	10	12	7	4	1	0	0	0	1	0	0	1	0	0	0	12	50	0.03
Lab 4	0	1	8	4	2	3	1	0	0	2	4	1	1	0	0	0	0	23	50	0.016
Lab 5	0	13	15	2	8	3	4	0	3	0	0	1	1	0	0	0	0	0	50	0.016
Total	1	34	56	19	24	12	6	0	4	3	5	7	2	1	1	4	5	68	252	0.016
Sma																				
Lab 1	0	0	0	1	16	17	9	1	0	0	1	2	0	1	0	2	0	0	50	0.125
Lab 2	0	0	0	0	1	20	19	10	0	1	0	0	0	0	1	0	0	0	52	0.125
Lab 3	0	0	0	1	1	13	13	8	6	2	1	0	2	2	0	0	1	0	50	0.125
Lab 4	0	0	0	0	3	24	9	4	4	0	1	0	0	2	1	1	1	0	50	0.125
Lab 5	0	0	0	0	0	13	15	10	2	1	0	1	2	1	2	1	1	1	50	0.25
Total	0	0	0	2	21	87	65	33	12	4	3	3	4	6	4	4	3	1	252	0.125
Sau																				
Lab 1	0	0	0	0	0	0	1	23	23	2	1	0	0	0	0	0	0	0	50	0.5 OR 1
Lab 2	0	0	0	0	0	0	0	10	38	7	0	0	0	0	0	0	0	0	55	1
Lab 3	0	0	0	0	0	0	0	0	2	37	5	0	0	2	2	1	0	1	50	2
Lab 4	0	0	0	0	0	0	0	1	15	32	2	0	0	0	0	0	0	0	50	2
Lab 5	0	0	0	0	0	0	0	0	13	35	2	0	0	0	0	0	0	0	50	2
Total	0	0	0	0	0	0	1	34	91	113	10	0	0	2	2	1	0	1	255	2
Aba*																				
Lab 1	0	0	0	0	0	0	0	0	4	24	9	2	3	4	4	0	0	0	50	2
Lab 2	0	0	0	0	0	0	0	5	18	29	6	0	0	0	0	0	0	0	58	2
Lab 3	0	0	0	0	0	0	0	1	9	31	6	1	0	0	0	1	0	0	49	2
Lab 4	0	0	0	0	0	0	0	0	4	29	9	3	0	1	2	0	1	1	50	2
Lab 5	0	0	0	0	0	0	0	1	20	16	6	0	0	0	0	1	0	0	44	1
Total	0	0	0	0	0	0	0	7	55	129	36	6	3	5	6	2	1	1	251	2

Eco, *E. coli*; Kpn, *K. pneumoniae*; Ecl, *E. cloacae*; Pmi, *P. mirabilis*; Sma, *S. marcescens*; Sau, *S. aureus*; Aba, *A. baumannii.* Lab 1, Huashan Hospital of Fudan University; Lab 2, Peking Union Medical College Hospital; Lab 3, Peking University First Hospital; Lab 4, Sir Run Run Shaw Hospital of Zhejiang University; Lab 5, Tongji Hospital of Tongji Medical College.

*For *A. baumannii*, the concentrations ranging from 0.004/0.002 to 256/128 mg/L, with drug ratio setting at cefotaxime:sulbactam = 2:1. For all other species, sulbactam was tested at a fixed concentration of 4 mg/L.

**TABLE 2:** Summary MIC statistics and final ECOFFs of cefotaxime–sulbactam and cefotaxime for the study species.

Species	Total number	Mode WT MIC	Range	MIC_50_	MIC_90_	Visual estimate ECOFFs (mg/L)	99% ECOFFs (mg/L)	Mode WT of cefotaxime alone (mg/L)	MIC_50_ of cefotaxime alone (mg/L)	MIC_90_ of cefotaxime alone (mg/L)	EUCAST ECOFF of cefotaxime alone (mg/L)
Eco	252	0.03/4	0.004/4 to > 256/4	0.25/4	128/4	0.25/4	0.25/4	0.06	8	> 256	0.25
Kpn	256	0.03/4	0.008/4 to > 256/4	0.125/4	128/4	0.25/4	0.25/4	0.06	0.12	> 256	0.25
Ecl	252	0.125/4	0.016/4 to > 256/4	0.25/4	2/4	0.5/4	0.5/4	0.25	0.5	32	1
Pmi	252	0.016/4	0.004 /4 to > 256/4	0.03/4	> 256/4	0.06/4	0.06/4	0.016	0.06	> 256	0.06
Sma	252	0.125/4	0.03/4 to > 256/4	0.125/4	2/4	1/4	1/4	0.25	0.25	64	/
Sau	255	2/4	0.25/4 to > 256/4	2/4	2/4	4/4	4/4	2	2	4	4
Aba	251	2/1	0.5/0.25 to > 256/128	2/1	4/2	4/2	4/2	2	2	32	64

### Establishment of ECOFF values

**FIGURE 1:**
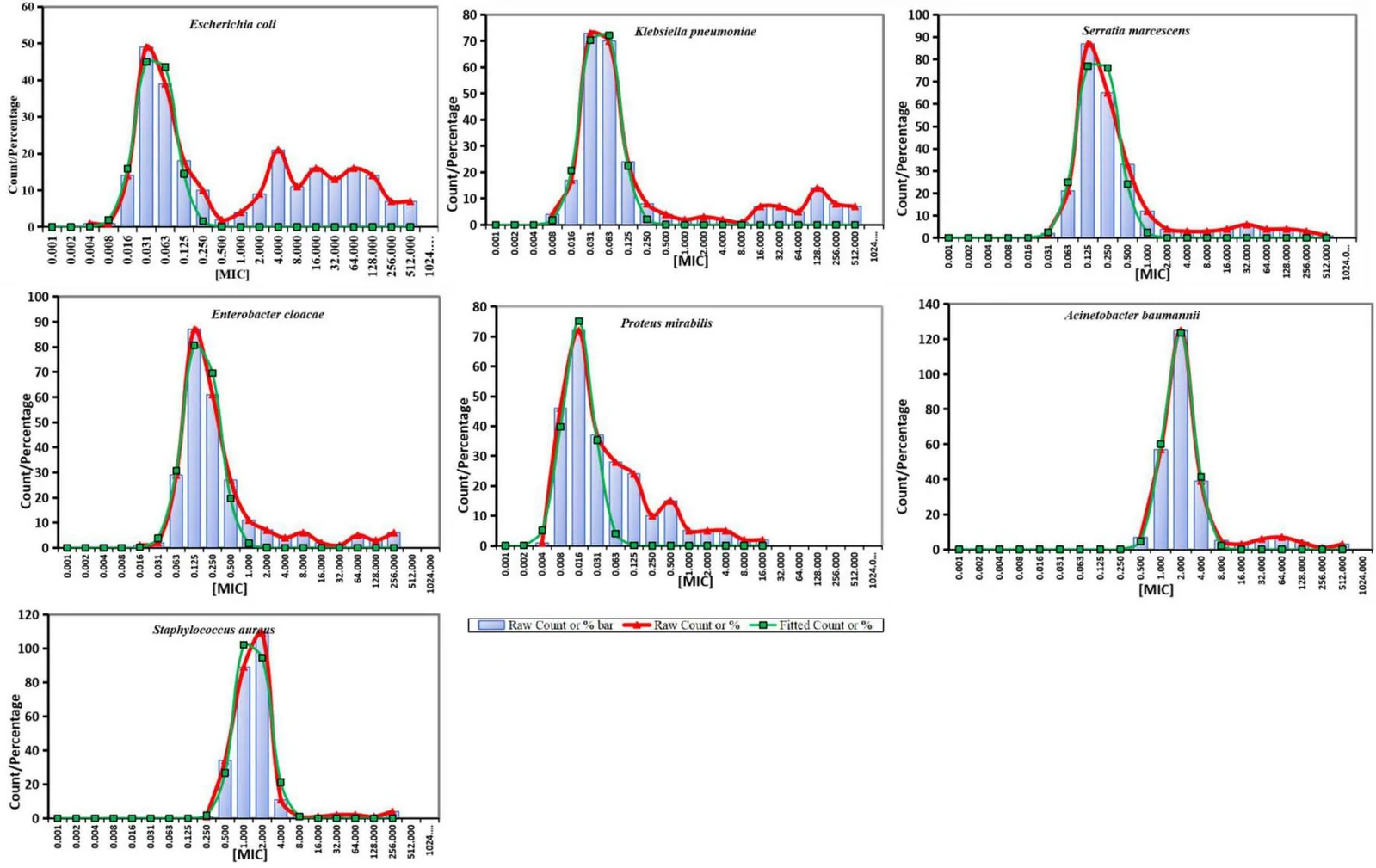
Non-linear regression fitting distribution of cefotaxime-sulbactam to seven species (Turnidge method). Red “Raw Count or %” represents measured MIC data, green “Fitted Count or %” represents simulated MIC data. The *X*-axis shows the cefotaxime concentration used to express the MIC of the cefotaxime-sulbactam combination. For *A. baumannii*, cefotaxime-sulbactam was tested at a cefotaxime:sulbactam ratio of 2:1; for the other species, sulbactam was tested at a fixed concentration of 4 mg/L. Histogram bars represent the number of isolates at each MIC dilution. The ECOFFs were estimated using ECOFFinder and visual inspection.

We calculated 95%, 97.5%, 99%, 99.5% and 99.9% endpoints, and selected 99% endpoints as defined by ECOFFs using ECOFFinder software (Figure 1). The final ECOFF values were determined by integrating the results from both ECOFFinder and visual inspection. For all seven species, the ECOFF assigned by visual inspection was identical to the ECOFFinder-derived 99% estimate. The ECOFF for *Enterobacter cloacae* was established at 0.5/4 mg/L, while *E. coli* and *K. pneumoniae* shared an ECOFF of 0.25/4 mg/L. The ECOFF for *S. marcescens* was 1/4 mg/L, *P. mirabilis* was 0.06/4 mg/L, *S. aureus* was 4/4 mg/L, and *A. baumannii* was 4/2 mg/L.

For *E. coli*, *K. pneumoniae*, *S. aureus* and *P. mirabilis*, the cefotaxime-sulbactam ECOFFs were identical to the corresponding EUCAST ECOFFs for cefotaxime alone. For *S. marcescens*, no established cefotaxime ECOFF is currently available in the EUCAST database. For *E. cloacae*, the combination ECOFF was one doubling dilution lower than the reported cefotaxime ECOFF. For *A. baumannii*, the cefotaxime-sulbactam ECOFF was 4/2 mg/L when tested at a 2:1 cefotaxime:sulbactam ratio, whereas the EUCAST cefotaxime-alone ECOFF for *A. baumannii* is 64 mg/L. Thus, the cefotaxime component of the combination ECOFF was markedly lower than the cefotaxime-alone ECOFF. However, this comparison should be interpreted cautiously because the *A. baumannii* assay used a variable sulbactam concentration in a fixed 2:1 ratio, whereas the other species were tested using a fixed sulbactam concentration of 4 mg/L.

## Discussion

This multicenter study systematically evaluated ECOFFs of cefotaxime-sulbactam against seven clinically relevant bacterial species using standardized broth microdilution methods. Overall, the MIC distributions of cefotaxime-sulbactam were broad across all species, reflecting the heterogeneous and representative susceptibility profiles observed in clinical isolates.

The accuracy of ECOFF determination relies heavily on the purity of the WT population analyzed. A major strength of our study design was the pre-selection of isolates based on defined susceptibility phenotypes to exclude known acquired resistance mechanisms, such as AmpC production (inferred by cefoxitin non-susceptibility) and carbapenemase production. This approach, as recommended by recent methodological guidance, minimizes the confounding effect of highly resistant subpopulations and allows for a more precise estimation of the upper limit of the true WT MIC distribution (CRyPTIC Consortium, 2022). This strategy does not guarantee a genetically pure WT population, but it improves interpretability of MIC distributions in the absence of comprehensive genotypic screening of all isolates. Furthermore, the rigorous external quality control program ensured that the MIC data aggregated from the five participating centers were harmonized and comparable, satisfying the EUCAST requirements for data aggregation (SOP 10.2).

The establishment of these ECOFFs provides a foundational tool for antimicrobial resistance surveillance and laboratory diagnostics (Hasan et al., 2026). For example, an Indian national surveillance data reported favorable activity of cefotaxime-sulbactam against common clinical isolates, which didn’t rely on empirical interpretation (Gondane and Pawar, 2023). By defining the upper MIC limit for the WT population, these values enable clinical laboratories to distinguish isolates that are likely to harbor acquired resistance mechanisms (non-wild-type, NWT) from those that do not. This is particularly important in the absence of clinical breakpoints. Importantly, the ECOFFs established for cefotaxime-sulbactam against *E. coli*, *K. pneumoniae*, *S. aureus* and *P. mirabilis* were identical to those reported for cefotaxime alone in EUCAST datasets. Clinically, this alignment provides a valuable reference: isolates that are WT for cefotaxime are highly likely to be WT for the cefotaxime-sulbactam combination. In contrast, the ECOFF for *A. baumannii* was 4/2 mg/L under the 2:1 cefotaxime:sulbactam ratio testing condition. This finding is consistent with the known intrinsic antibacterial activity of sulbactam against *Acinetobacter* spp (Penwell et al., 2015). The observed MIC distribution and ECOFF for *A. baumannii* reflect the combined activity of cefotaxime and concentration-dependent sulbactam exposure. For *E. cloacae* complex, the one-doubling-dilution difference between the cefotaxime-sulbactam ECOFF and the cefotaxime-alone ECOFF falls within the accepted technical variation of broth microdilution testing and should not be interpreted as evidence of AmpC inhibition by sulbactam. This highlight the importance of resistance surveillance (Ahmed Hasan et al., 2025).

In this study, the presence of highly resistant strains did not affect the establishment of ECOFF for all species. Both ECOFFinder and visual methods effectively distinguished wild-type from non-wild-type strains. For *P. mirabilis*, 118 isolates exhibited MICs above the established ECOFF (0.06/4 mg/L) and were classified as non-wild-type. Among them, 96 available isolates were subjected to WGS, revealed frequent carriage of multiple β-lactamase genes. Fifty strains simultaneously carried *OXA-1* and *CTX-M-65, CTX-M-55* or *TEM-1*, while 6 strains carried only *OXA-1*. In addition, 28 strains carried one or more extended-spectrum β-lactamase genes, whereas 12 strains carried did not carry identifiable acquired β-lactamase genes based on the resistance gene annotation pipeline used in this study. These findings are consistent with international genomic data showing that acquired β-lactamase genes, particularly *bla*_*CTX–M*_, *bla*_*TEM*_, and OXA-type determinants, are major contributors to cephalosporin resistance in *P. mirabilis* (Zhang et al., 2026). One possible resistance mechanism is as follows: OXA-1 is a class D β-lactamase capable of hydrolyzing cephalosporin β-lactam antibiotics (including cefotaxime) and exhibits resistance to traditional β-lactamase inhibitors such as sulbactam (Gatermann and Marre, 1991; Papp-Wallace et al., 2023). Moreover, in strains carrying multiple β-lactamase genes, overexpression of β-lactamases may further diminish the inhibitory capacity of sulbactam, thereby contributing to elevated MICs (Shaaban et al., 2022; Tamma et al., 2022). In a minority of isolates lacking identifiable resistance determinants, mutations leading to the loss or reduced expression of outer membrane porins can significantly impede the entry of both cefotaxime and sulbactam into the bacterial cell. The upregulation of multidrug efflux pump systems may lead to the resistance of cefotaxime-sulbactam, too.

Several limitations should be acknowledged. First, different testing configurations were used for *A. baumannii* and the other species, which limits direct interspecies comparison. Second, wild-type population was defined and enriched solely on the basis of phenotypic MIC distribution criteria, without independent molecular confirmation of the absence of resistance determinants in all isolates. Third, this study focused exclusively on *in vitro* susceptibility testing and did not incorporate pharmacokinetic/pharmacodynamic (PK/PD) analyses. As a result, the ECOFFs established here cannot be directly translated into clinical breakpoints. Future studies integrating PK/PD modeling and clinical outcome data are required to define optimized dosing regimens and establish interpretive breakpoints for cefotaxime-sulbactam. Nonetheless, the ECOFFs presented here provide an essential foundation for AST interpretation and for future breakpoint development.

## Conclusion

This study included 1,770 isolates collected from five laboratories across four cities in China. Through multicenter microbroth dilution experiments, we established the MIC ECOFF values for cefotaxime-sulbactam against *E. coli*, *E. cloacae*, *K. pneumoniae*, *P. mirabilis*, *A. baumannii*, and *S. aureus*. These findings provide a foundation for future PK/PD analyses and determining the clinical breakpoints of cefotaxime-sulbactam, thereby enhancing the therapeutic options available to clinicians and supporting more informed clinical decision-making in the treatment of bacterial infections.

## Data Availability

The original contributions presented in this study are included in this article/supplementary material, further inquiries can be directed to the corresponding author.

## Footnotes

1
https://github.com/tseemann/shovill

2
www.eucast.org/mic_distributions_and_ecoffs
